# Microbial Communities in Permafrost Soils of Larsemann Hills, Eastern Antarctica: Environmental Controls and Effect of Human Impact

**DOI:** 10.3390/microorganisms8081202

**Published:** 2020-08-07

**Authors:** Ivan Alekseev, Aleksei Zverev, Evgeny Abakumov

**Affiliations:** 1 Department of Applied Ecology, Faculty of Biology, Saint Petersburg State University, 199034 Saint Petersburg, Russia; azver.bio@gmail.com (A.Z.); e_abakumov@mail.ru (E.A.); 2 All-Russian Research Institute for Agricultural Microbiology, 196608 Saint Petersburg, Russia

**Keywords:** extremophiles, Antarctica, soil parameters, human impact, microbial communities

## Abstract

Although ice-free areas cover only about 0.1% of Antarctica and are characterized by harsh environmental conditions, these regions provide quite diverse conditions for the soil-forming process, having various physical and geochemical properties, and also assuring different conditions for living organisms. This study is aimed to determine existing soil microbial communities, their relationship with soil parameters and the influence of anthropogenic activity in Larsemann Hills, Eastern Antarctica. The soil microbiome was investigated at different locations using 16S rRNA gene pyrosequencing. The taxonomic analysis of the soil microbiomes revealed 12 predominant bacterial and archaeal phyla—Proteobacteria, Actinobacteria, Acidobacteria, Chloroflexi, Gemmatimonadetes, Verrucomicrobia, Planctomycetes, Bacteroidetes, Armatimonadetes, Firmicutes, Cyanobacteria, Thaumarchaeota. Some specific phyla have been also found in sub-surface horizons of soils investigated, thus providing additional evidence of the crucial role of gravel pavement in saving the favorable conditions for both soil and microbiome development. Moreover, our study also revealed that some bacterial species might be introduced into Antarctic soils by human activities. We also assessed the effect of different soil parameters on microbial community in the harsh environmental conditions of Eastern Antarctica. pH, carbon and nitrogen, as well as fine earth content, were revealed as the most accurate predictors of soil bacterial community composition.

## 1. Introduction

Although ice-free areas cover only about 0.1% of Antarctica, these regions provide quite diverse conditions for the soil-forming process, having various physical and geochemical properties, and also providing different conditions for living organisms. In Antarctica, microbial communities are generally the dominant biomass component of terrestrial ecosystems, also they control most of the biological flux of carbon, nutrients and energy [1,2]. Soil is an important component of Antarctic ecosystems, since it determines their sustainability and serves as a habitat for living organisms. Soil microbiomes play an essential role in the development of soil profiles and the implementation of soil biochemical processes. Soil genesis is closely related to the activity and development of its microbiome [3]. Microorganisms play a key role in ensuring the cycling of the main nutrients during the decomposition of organic material and in the formation of organic matter in the soil.

Antarctica weakly obeys the general geographic law of latitudinal zoning. Remote ice-free areas (oases) are isolated from each other and have no biological or even climatic connection, so they are more like islands in the ocean. According to Bockheim and Hall [4], three climatic zones can be distinguished in Antarctica: Subantarctic (including the South Shetland Islands), Antarctic coastal and Antarctic continental zones.

Eastern Antarctica oases are characterized by climatic extremality, which in turn determines the specificity of soil formation. Acute lack of moisture, ultraviolet radiation, sharp temperature changes and strong winds significantly reduce the primary production of organic matter and the formation of organogenic horizons on the surfaces of loose and rocky substrates that prevail in Eastern Antarctica oases [5]. An important feature of the landscapes of the oases is that most of the biomass is concentrated beneath the mineral surface, which greatly changes the vertical organization of the soil profile. In these extreme habitats, the leading role in soil formation belongs to the most adapted fungi and bacteria [6].

Investigation of microbial communities in extreme environments have been actively discussed in recent decades. Many researchers indicated a significant diversity of microorganisms in Antarctic soils and rocks [1,7,8,9]. Special attention has been given to investigation of cryptoendolithic systems to reveal fungal and bacterial community structure in harsh environments of different regions of Antarctica [10,11,12,13]. Previous studies of microbial communities in soils have been mainly conducted in the Transantarctic region of the Victoria Land, Antarctic Peninsula and surrounding Islands. According to these investigations, microbial communities in Western Antarctica are dominated by several bacterial phyla, such as Proteobacteria, Bacteroidetes, Actinobacteria, Cyanobacteria, Verrucomicrobia, Thermotogae, Fibrobacteres, Deinococcus-Thermus and Chlorobi [14,15,16,17]. At the same time, the microbial communities of Eastern Antarctica coastal oases remain less investigated. Moreover, relatively little attention has been shown towards soil microbial communities, although they could serve to enhance our knowledge on life adaptation strategies under such harsh environmental conditions. It has been shown that soil bacterial communities in Eastern Antarctica contain a significant amount of small filtering bacterial forms, which is considered as an adaptation to the harsh climatic conditions of Antarctica [6,8]. A role of human impact on soil bacterial diversity in Eastern Antarctica has been rarely investigated as well. Previously, anthropogenic factor were found to affect both soil geochemical properties and soil bacterial diversity from sites around Casey Station [7]. Nowadays, anthropogenic impact on the natural ecosystems of both Western and Eastern Antarctica is expressed not only in contamination, but also in various physical disturbances, such as soil over-compaction caused by mechanical impact, soil surface consumption by a waste disposal and changes in thermal regime (especially in the areas of polar stations and their surroundings).

The human disturbance footprint for the entire Antarctic continent has recently been calculated [18,19,20]. Effective realization of environmental protection measures is vital for saving unique ecosystems of the sixth continent as well as for appropriate compliance of the Antarctic Treaty’s fundamental principles. These measures include environmental impact assessment and permanent monitoring of various components in natural ecosystems, such as soil. Due to the unique geological structure, biological diversity and increasing rates of human activity (e.g., tourism activity, chemical contamination and physical disturbance of surface and permafrost), the Antarctic Specially Managed Area (ASMA-6) has been designated in the Larsemann Hills. Additionally, the Antarctic Specially Protected Area (ASPA № 174) has been established in the area of Stornes Peninsula to “protect the outstanding geological features of this area, specifically the rare mineral occurrences and the highly unusual host rocks in which they occur” [21].

It should be concluded that soil microbiological studies in Antarctica are still lacking in regard to investigation of taxonomic composition and functional diversity of microbiome. Although some research revealed the Antarctic microbiome as unique [22,23], other studies showed microbiome composition similar to other regions of the Earth [15,24]. Moreover, increased rates of anthropogenic forcing (tourism activities, logistic load, functioning of the numerous scientific stations) require more attention to be paid, with specific studies dedicated to revealing the possible changes in microbiome due to anthropogenic transformation. Modern pyrosequencing techniques allow not only to identify the culturable bacteria in various Antarctic environments, but also delineate the wide spectra of unculturable bacteria associated with humans. In this respect, using the next-generation sequencing techniques to study the microbiome of soils in vicinities of Antarctic stations are especially relevant, since these data are still poor [25].

Moreover, comprehensive knowledge of the microbial community is needed for studying the fundamental problem of mineral substrate transformation under extreme climatic conditions of Antarctica. This is especially relevant in regards to studying the initial soil-forming process and finding the modern analogues of Precambrian soils [26].

The aim of this work was to assess the microbial communities in soils of Larsemann Hills, Eastern Antarctica. The objectives of this work were:

- To characterize taxonomic diversity and soil-physical properties of soils of Larsemann Hills;
- To assess soil microbiome composition and its variability across landscapes of Larsemann Hills, as well as the role of anthropogenic factor effect on soil microbiome structure and composition;
- To reveal the relationship between soil microbiome composition and diversity with soil physical–chemical properties.

## 2. Materials and Methods 

**Figure 1:**
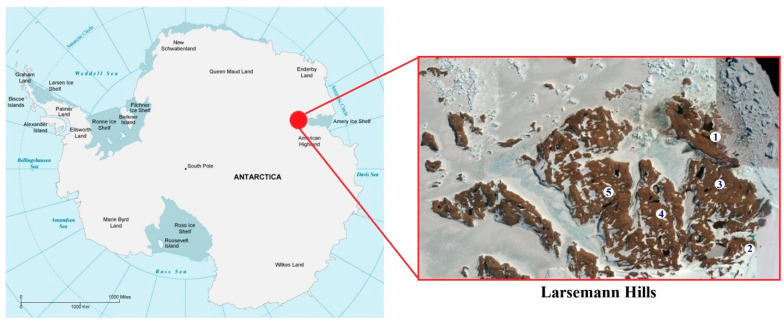
Map of the study area. Anthropogenically-affected sites: 1—Progress 1; 2—Progress 2. Near-natural sites: 3—Lars 1; 4—Lars 2; 5—Lars 3. *Image credit: ©* *Google Earth* *2010.*

Field work and soil sampling were conducted during the 63rd Russian Antarctic Expedition (December 2017–April 2018). Investigation sites were located in both near-natural and anthropogenically-affected areas of Larsemann Hills, Eastern Antarctica (Figure 1).

The main climatic parameters of investigation area are summarized in Table 1. The main limiting factor for soil formation in Eastern Antarctica oases is the lack of moisture. Melting snow in the summer months is the determining regulator of bio- and pedological diversity [5]. In conditions of almost complete absence of liquid precipitation, low air humidity existence of living is supported only by melting water from snow patches.

**Figure 2:**
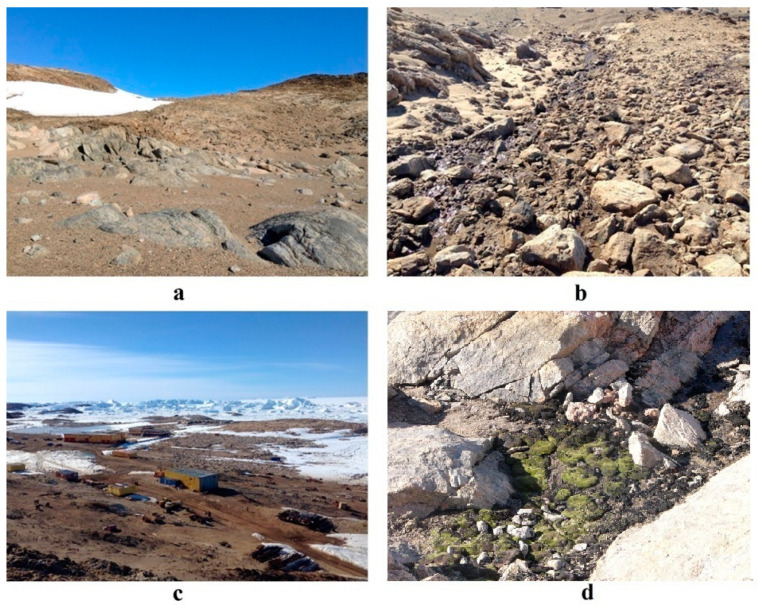
Landscape diversity of Larsemann Hills. (**a**)—Larsemann Hills (high flank with dry conditions); (**b**)—Larsemann Hills (valley bottom with meltwater flow); (**c**)—Larsemann Hills (view on the Progress Station); (**d**)—Larsemann Hills (wind shelter on the hillslope).

Larsemann Hills oasis is deeply dissected by short (up to 1 km) valleys, which formed along the lineaments as a result of glacial erosion [28]. Inter-mountain valleys are the most important structural element of the oasis, because they serve as a landscape background for wet valleys formation (Figure 2). The main feature of the climate of this oasis is persistent, strong winds blowing from the north-east during most of summer days. Geologically this oasis mostly consists of gneisses, crystalline shale. In austral summer, many valleys in Larsemann Hills are waterlogged owing to melting glaciers and thawing of ice-rich frozen ground, thus the soil formation is strongly affected by hydrological and cryogenic factors [28]. Permafrost table usually lies at the depth of 95–100 cm [29]. Larsemann Hills are characterized by relatively homogenous flora, and mainly limited by communities of lichens, mosses and algae [28]. Taxonomic analysis revealed 7 moss species (*Bryum pseudotriquetum* is dominant), 56 lichen species (*Umbilicaria decussate* and *Buellia frigida* are dominants), about 200 taxa of terrestrial algae and 100–200 taxa of microscopic fungi.

The area of Larsemann Hills is potentially at high environmental risk, since 4 research stations are present in Larsemann Hills research: Progress station (Russia), Bharati station (India), Law Base (Australia) and Zhongshan (China). The Progress station has a stationary helicopter base, wintering complex consisting of office and residential buildings, power station, diesel electrostation, workshop and garage. There is a warehouse for open storage of disposed waste.

### Soil Sampling and Laboratory Analyses

Topsoil samples have been collected from described sites during the field work in December 2017–February 2018. Each of the studied site is 50 × 50 m and characterized by 3 sampling points. Soils were sampled from 20 × 20 cm soil pits from different depths. The samples were stored in double sterile plastic bags, labelled and transported to the laboratory. The samples were air-dried at room temperature, separate from roots and debris, and passed through a 2 mm plastic sieve prior to chemical analysis. The pH was determined using a pH-meter and exchangeable and hydrolytic acidity was measured by titration with KCl and CH3COONa, respectively (soil:solution ratio 1:2.5). Soil basal respiration was evaluated in laboratory closed chambers by CO2 concentrations in an alkaline solution that was saved in a plastic container during the incubation process for 10 days. Carbon and nitrogen content was measured using the CHN elemental analyzer (Elementar Analyse Systeme GmbH, Vario MAX). Particle size distribution analysis was performed with the pipette-sedimentation method [30]. All physical–chemical and biological parameters were analyzed at least in triplicate (n = 3–6). Calculated mean values are provided with standard deviations (a ± b).

Classification of soils was carried out according to World Reference Base for Soil Resources (WRB) [31]. It should be noted that issues related to the classification of Antarctic soils are still debatable, disputes are being held regarding the validity of the distinguishing of certain types of soils. The detailed description of investigation sites are given in Table 2.

Topsoil material for DNA extraction were not ground before analyses. Samples were frozen in the field and transported to the laboratory. DNA was extracted from 5.0–7.0 g of soil material using the PowerSoil DNA Isolation Kit (Mobio Laboratories, Solana Beach, CA, USA), which included a bead-beating step, according to the manufacturer’s specifications. Homogenization of the samples was performed using Precellys 24 (Bertin Corp, Rockville, USA) at 6.5 m/s, twice for 30 s. The purity and quantity of DNA were tested by electrophoresis in 0.5 × TAE buffer on 1% agarose. DNA concentrations were measured at 260 nm using a SPECTROStar Nano (BMG LABTECH, Ortenberg, Germany). The average DNA yield was 2–5 μg DNA, with concentrations between 30 and 50 ng/μL. The purified DNA templates were amplified with universal multiplex primers F515 5′-GTGCCAGCMGCCGCGGTAA-3′ and R806 5′-GGACTACVSGGGTATCTAAT-3′ [32] targeting the variable region V4 of bacterial and archaeal 16S rRNA genes. Each multiplex primer contained the adapter, 4-bp key (TCAG), 10-bp barcode and primer sequences. The expected length of the amplification product was 400 bp. Sequencing of the amplicon libraries was carried out using Illumina MiSeq (pair-ended reads) in the Centrum ‘Genomic Technologies, Proteomics and Cell Biology’ (All-Russia Research Institute for Agricultural Microbiology, Russia). The raw sequences were processed using QIIME [33]. Preliminary quality filtering (with parameters minimal length 180 and sliding window 4:16) of the raw reads was performed using TRIMMOMATIC software [34]. To reduce sequencing errors, the multiplexed reads were first filtered for quality and grouped according to barcode sequences. Sequences were omitted from the analysis if they were less than 200 bp, had a quality score of less than 25, contained uncorrectable barcodes, primers, ambiguous characters or a homopolymer length equal to or greater than 8 bp. All non-bacterial ribosomal sequences and chimeras were also removed from the libraries. Chimeras were removed by using chimera_slayer.py script, incorporated in QIIME. A representative set of sequences was chosen by selecting the most abundant sequence from each Operational Taxonomic Unit (OTU). Representative sequences from each OTU were subjected to an RDP naïve Bayesian rRNA Classifier [35], with a confidence level of 97%, and aligned using a PyNast algorithm and SILVA database [36]. Aligned sequences were used to build a distance matrix with a distance threshold of 0.1 and phylogenetic tree necessary for downstream analysis.

To estimate alpha diversity, OTUs number as well as several indices—Shannon’s index, Simpson’s index and phylogenetic diversity—were calculated. The t-test was performed to verify the observed differences. The abundances of OTUs were compared between samples by calculating the median relative change values for all groups of replicates. A positive median indicates an increase in abundance, whereas a negative median can be considered as evidence for a decline in abundance. A basic permutation test was used to infer significance, whereas a jackknife-like resampling approach was applied to test the stability of median estimates.

Statistical analyses were performed using JASP analytical software (JASP TEAM, Amsterdam University).

## 3. Results and Discussion

### 3.1. Soil Physical–Chemical Properties

The main physical–chemical parameters of investigated soils are summarized in Table 3. Values of pH in all studied soils were moderately acidic to slightly neutral. This is due to the absence of the salt accumulation from the ocean acid to the neutral nature of parent materials. The soils investigated contained different amounts of total organic carbon (TOC), which ranged from 0.54 to 1.64%, being the highest in sub-surface algal–mineral and moss–algal–mineral horizons in natural sites. Therefore, we can summarize that soils of Larsemann Hills have different levels of carbon and nitrogen accumulation, being lower than reported for Maritime Antarctica [37,38,39,40]. Additionally, carbon to nitrogen (C:N) ratio varied between 10.48 and 16.33. A narrow range observed in some topsoil horizons could be explained by the presence of well-decomposed organic matter (mainly peat material). This has been also described for soils in Eastern Antarctica previously [26]. Basal respiration (BR) rates ranged from 0.006 ± 0.001 to 0.150 ± 0.010 mg g^−1^ day^−1^, which is in agreement with results previously published for coarse soils of Antarctica [41,42]. Higher values of BR were observed at sub-surface horizons with hypolithic algae–bacterial communities. However, it should be noted that higher basal respiration rates could be found in soils under strong influence of birds (penguin rockeries, nesting sites of penguins and flying birds), which were described previously for both Western and Eastern Antarctica [43,44,45]. The percentage of fine earth ranged between 18.99 to 31.98% in studied soils of Larsemann Hills, with highest values in sub-surface horizon with more favorable conditions for fine earth formation.

The main limiting factor for soil formation in Eastern Antarctica oases is the lack of moisture. Melting snow in the summer months is the determining regulator of bio- and pedological diversity [5,46]. In conditions of very low liquid precipitation, low air humidity existence of living organisms is supported mostly by melting water from snow patches. However, usually soil parameters of Eastern Antarctica do not show any clear trend in profile distribution and could be largely affected by microclimate effect and local-scale variability [47].

### 3.2. Microbiome Composition of Studied Soils

**Figure 3:**
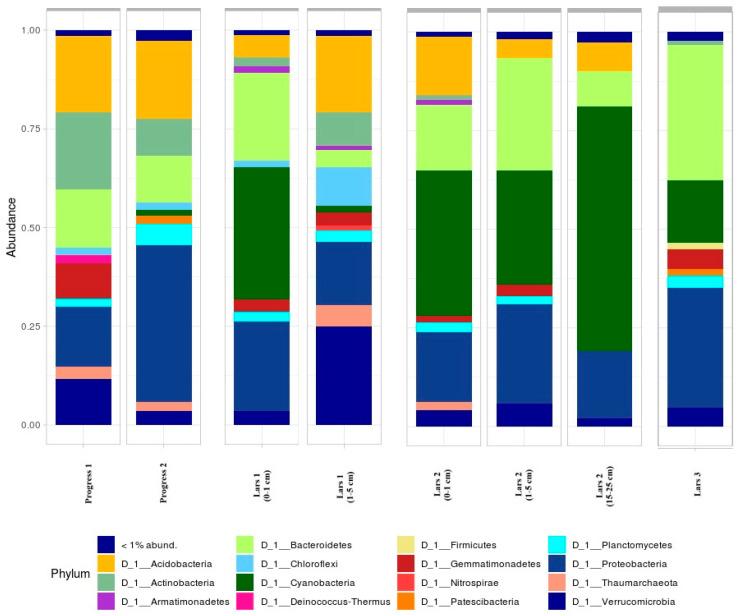
Taxonomic composition at phylum level of soil microbial communities of investigated soil samples.

The taxonomic analysis of the soil microbiomes revealed 12 major bacterial and archaeal phyla-Proteobacteria, Actinobacteria, Acidobacteria, Chloroflexi, Gemmatimonadetes, Verrucomicrobia, Planctomycetes, Bacteroidetes, Armatimonadetes, Firmicutes, Cyanobacteria and Thaumarchaeota, which constituted the majority (>96% of sequences in amplicon libraries) (Figure 3). The rest of the identified phylum accounted for less than 0.01% of the total.

To evaluate the alpha-diversity of the soil microbiomes, OTUs were identified and several indices (Shannon, Simpson and Phylogenetic diversity) were calculated (Table 4). For OTUs that significantly differed between the samples, further taxonomic identification using the RDP and GenBank databases were conducted. Only the OTUs that had more than 98% identity to the nearest homologous sequence in GenBank (the sequences with known taxonomic names were used) were included in the analysis. A significant proportion of these OTUs were homologous to species found in polar environments (both in Arctic and Antarctic regions), among which were samples from glacial forefield in Larsemann Hills [22] and Fildes Peninsula, King George Island [25].

OTUs number varied significantly among studied soil samples (from 310 in transitional horizon of Lars 2 site to 760 in surface Progress 2 sample). Shannon’s index mean values ranged from 4.02 to 5.31 in studied samples. Similar values has been reported previously for different glacier forefield areas of Larsemann Hills [22]. Lower values of Shannon’s index have been observed in soils (3.3) and endoliths (2.8) of McKelvey Valley, McMurdo Valley [48]. Other investigations on endolithic microbial communities also reported lower values of this index [49,50]. However, significantly higher values of the Shannon’s diversity index have been reported for the Maritime Antarctica soil microbiome [25].

The highest diversity was found in the Progress 2 sample, which corresponds to the topsoil from the field base facilities. The soil surface is experiencing an active physical disturbance during the summer season, when numerous scientific operations are carried out. The site is also affected by vehicles since the site is used for logistical support for Progress airfield. High alpha-diversity at this site might be also caused by high amount of available moisture, since the site is located in wet valley accumulating water from melting snow patches and precipitation. The highest phylogenetic diversity (PD_tree) was also found in topsoil sample from Progress 2 site. Therefore, we can suppose that the higher Shannon index here is caused by anthropogenic inputs. Simpson’s diversity index varied less significantly among studied soil samples (from 0.945 in Lars 2 transitional horizon to 0.985 in topsoil from anthropogenically-affected site at Progress station). These values are higher compared to previously reported data reported for different endolith types from the McKelvey Valley [48], Miers Valley [49], different locations in McMurdo Valleys [50] as well as for soils of the glacier forefield in Larsemann Hills [22].

**Figure 4:**
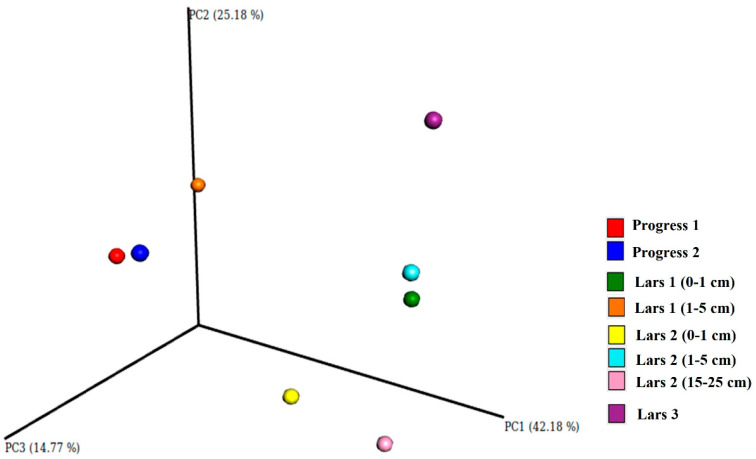
PCoA analysis of weighted UniFrac distance matrix representing β-diversity of the prokaryotic communities. PC1, PC2 and PC3 are the main coordinate axes of the multidimensional space, with the values of the explained variance (percentage).

A clear trend of decreased alpha-diversity in deep soil horizons (where no features of macroscopic life is observed) was revealed for near-natural sites. The lowest alpha-diversity values were observed for transitional BC horizons of Lars 2 profile (Figure 4). However, this is not always true for soil profiles in Eastern Antarctica—especially in bottoms of wet valleys, where cryogenic processes (cryoturbation, patterned ground formation, frost sorting) are very active. A characteristic feature of Eastern Antarctica—presence of life mainly in sub-surface soil layers, which are not under direct influence of harsh conditions (UV-radiation, strong winds, low temperatures)—lead to heterogeneity of profile distribution of soil organisms. The mineral substrate in soils serves as a protector for microorganisms.

In general, soils of sub-surface layers at both anthropogenic and near-natural sites showed greater evenness and richness compared to surface and deep soil horizons. At the same time, the area under anthropogenic influence (Progress 2 site) showed the highest richness, which indicates that human impact could lead to introducing some bacterial species into Antarctic soils. Ordination analyses of weighted UniFrac distances for bacteria supported these trends in alpha diversity (Figure 4).

The content of microorganisms in soils of Eastern Antarctica was previously counted [6]. Authors investigated microbial biomass in soils of Thala and Larsemann Hills and noticed a significant amount of bacterial filtering forms, which by their small size might indicate an adaptation to severe environmental conditions of Eastern Antarctica coastal oases. It was also reported less biomass in soils of the Thala Hills, which were caused by less developed moss cover on the soil surface. This coincides with our data of higher bacteria abundance in soils with developed moss cushion under gravel pavement in near-natural profiles, where sub-surface horizons were examined.

### 3.3. Anthropogenically-Affected Sites

Progress 1 (0–1 cm) sample is characterized by very high percent of Cyanobacteria phylum (33%) in its composition (Figure 3). Additionally, this sample is dominated by Proteobacteria (22%) и Bacteroidetes (21%) phyla. However, the average level of alpha-diversity was observed for this sample. Progress 2 sample is characterized by very high alpha-diversity. Interestingly, a high proportion of Chloroflexi and Verrucomicrobia in the microbial community was noticed. The Deinococcus-Thermus group was found only in one of eight samples investigated—Progress 1 sample. Representatives from this group have been previously found in numerous Antarctic desert soil environments with dry and low-productive conditions [51,52,53]. They are characterized by high tolerance to ultraviolet radiation and desiccation, so this allows them to be found in the surface horizon (Progress 1 sample).

Both topsoil samples collected from anthropogenic sites are characterized by relatively high alpha-diversity. These samples are clustered together according to their beta-diversity when using principal coordinate analysis (PCoA, Figure 4). At the same time, some significant variability in taxonomic composition of microbiome composition was noticed between two “anthropogenic” samples. Progress 2 sample is dominated by the Proteobacteria phylum, whereas Progress 1 is also characterized by the presence of the Gemmatimonadetes phylum and a higher amount of bacteria from the Verrucomicrobia phylum. The Patescibacteria phylum, although not with high abundance, was observed in the Progress 2 sample as well. 

Proteobacteria have been previously found in Antarctic soils [25,54,55]. However, their habitats have been described to vary significantly in regards to changing nutrients and water availability, temperature regime and soil properties. At the same time, Proteobacteria was reported to be more abundant in highly productive soils rather than low productivity [52]. 

The Gemmatimonadetes phylum are to be found in various geographical locations, including Antarctic soils and cryoconite holes [56,57]. They have a diverse functional and metabolic potential, which allow them to colonize numerous ecological niches, including cold-affected habitats in Eastern Antarctica oases. However, only few isolates have been described previously [22,58], so their role in the soil microbiome remain unclear.

The Actinobacteria phylum, which is common for both anthropogenic samples examined, could be found in various cold-affected habitats worldwide [22,48,49,50,59]). Representatives of this phylum successfully colonize different habitats (including extreme environments), since they are able to metabolize a wide range of substrates as sole carbon source and release simple carbon compounds as compatible solutes that protect the organisms against freezing [2,51].

It has been previously discussed that numerous natural and anthropogenic factors influence soil fertility and microbial community and have to be considered when interpreting the soil microbiome parameters [60,61]. In this work we discuss only some of them. In Antarctic environments soil microorganisms as well as soil formation per se are mostly limited by deficit of moisture (rather than the low temperature), which is under direct influence of micro-scale variability.

### 3.4. Near-Natural Sites

Soil samples from the second “near-natural” soil profile (Lars 2) are similar in regards to their taxonomic composition (Figure 3). Cyanobacteria, Bacteroidetes, Proteobacteria and Acidobacteria are the most abundant phyla for the soil profile. Cyanobacteria have a significant portion in samples’ composition (up to 61%). This; however, probably causes the decrease in levels of alpha-diversity for the Lars 2 (15–25 cm) sample. According to beta-diversity analysis, these samples; however, do not form a common cluster although they are placed on the same axis of the PCoA graph (Figure 4). Thus predicting that there would be pronounced differences in the community structure at the lowest taxonomic levels. The observed differences in the structures of the microbiome in the soils examined might be explained by the fluctuations in their taxonomic structures at the family, genus and species levels.

It has been previously discussed for Larsemann Hills soils that high Cyanobacteria abundance are usually associated with seasonal meltwater from melting snow patches [62]. It has been noticed that organic material in such soils are usually represented by “black material” and higher organic carbon contents compared to dry high flanks of the valley. Higher abundance of microorganisms in such “moist” horizons could indicate that these soils have undergone longer development and could represent a more “matured” genetic stage of soil formation.

Relatively high abundance of Acidobacteria was observed in the sub-surface horizon of wet valley (Lars 2 1–5 cm). This, together with higher abundance of Acidobacteria in the transitional oximorphic horizon (15–25 cm) of the Lars 2 profile, indicate more “oligotrophic” conditions of both the wet valley and seasonal melt flow could support the development of an Acidobacteria-related community, since these bacteria could live under extremely low nutrient conditions and show very slow metabolic growth rate. This is also in line with data reported by Ward et al. [63], who showed that Acidobacteria could tolerate significant fluctuations in soil hydration. It has been also shown previously that Acidobacteria are quite common in different environments of the Antarctic continent [22,25,50,53,59], although their role remains poorly understood due to lack of experimental data. Although the Nitrospirae phylum was observed only in one investigated sample—Lars 1 (1–5 cm)—its role in primary productivity of unique ecosystems of Eastern Antarctica could not be underestimated. This further provides an evidence of higher productivity in sub-surface soil horizons rather than at surface.

Lars 3 sample is characterized by relatively low level of alpha-diversity. Taxonomically, the microbial community is dominated by Proteobacteria (31%), Bacteroidetes (34%) and Cyanobacteria (16%) phyla. Absence of bacteria from Acidobacteria might be also observed. This is caused by very low available moisture at this site. The high abundance of Bacteroidetes at this poorly developed habitat play an important role, since representatives of this phyla support initial soil formation contributing to biological weathering through degrading of polymers and producing extracellular enzymes [48,64,65]. The Patescibacteria phylum is also found in this sample. This phylum together with other related candidate phyla have been recently proposed to the CPR supergroup [66], and are characterized by small genomes reduced metabolic capabilities that likely have prevented their cultivation so far [66,67]. Metagenomic features of the Patescibacteria phylum suggest a strictly anaerobic fermentation-based lifestyle, which might give specific advantages for surviving and thriving in harsh permafrost environments (which are likely oxygen limited).

It has been widely discussed among Antarctic microbiologists that Proteobacteria and Deinococcus-Thermus bacterial phyla are well-adapted to harsh environmental conditions of Antarctica, while Acidobacteria and Bacteroidetes have been described as largely inactive [68,69,70]. Moreover recent studies suggested that Acidobacteria phylum representatives, which are often found in various Antarctic environments, could survive in different dormant forms [71].

**Figure 5:**
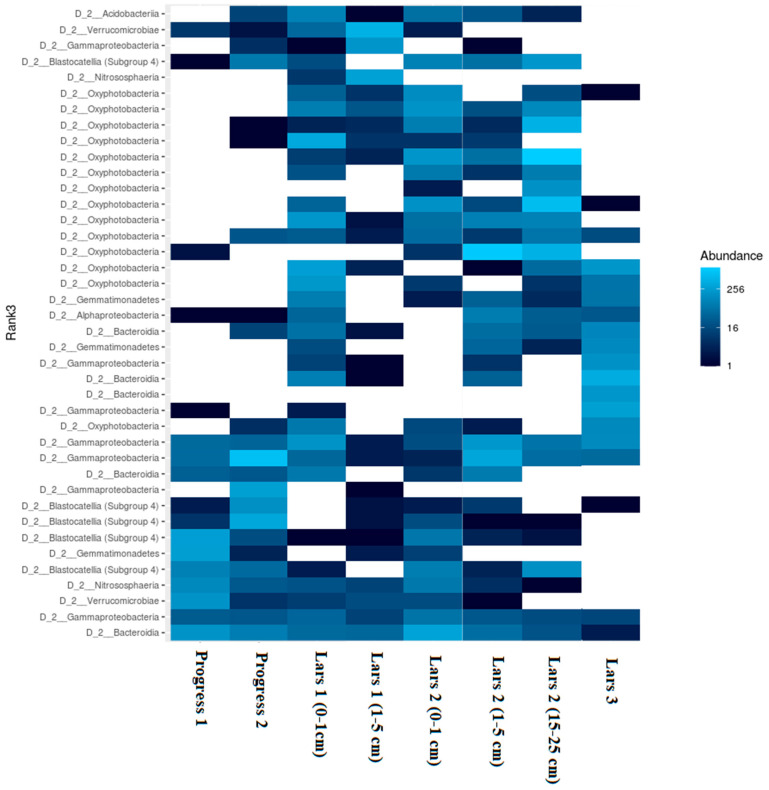
Heatmap of the most abundant phylotypes in investigated soil samples.

To get better insight into the differences of the soil bacterial community among the soils examined, heatmap analysis of the most abundant OTUs was applied (Figure 5). As shown in the heatmap, the abundance of dominant OTUs differed among the soil samples investigated. As it is seen from the heatmap, samples from anthropogenic areas substantially differed from near-natural sites in regards of abundance of different soil microbial groups. Progress 1 sample was dominated by Bacteroidia, Verrucomicrobia, Blastocatellia and Gemmatimonadetes classes. Progress 2 sample was dominated by Gammaproteobacteria, Blastocatellia and Bacteroidia classes. Surface sample Lars 1 (0–1 cm) was characterized by high abundance of Oxyphotobacteria, Gemmaproteobacteria and quite high abundance of Acidobacteriia classes. Surface soil horizons from the Lars 2 profile were characterized by high abundance of Bacteroidia, Blastocatellia and Oxyphotobacteria classes. The surface Lars 3 sample was dominated by Bacteroidia, Oxyphotobacteria and Gammaproteobacteria. Sub-surface horizons from Lars 1 and Lars 2 soil profiles were found to be dominated by different classes. The Lars 1 (1–5 cm) sample Verrucomicrobiae, Acidobacteriia and Nitrososphaera classes were the most abundant, whereas Lars 2 (1–5 cm) sample was dominated by Gammaproteobacteria and Oxyphotobacteria classes.

### 3.5. Relationship between Soil Microbiome and Soil Physical–Chemical Properties

Results of previous studies showed that soil pH and nitrogen content are the most important contributors to the variation in soil microbial communities and are considered as good predictors of bacterial community composition [60,72,73]. pH has been described as the main environmental factor to determine soil microbial community composition not only across biomes, but also for individual soil types [73,74]. The authors explained a strong influence of pH on the bacterial community composition by the narrow pH ranges for optimal growth of bacteria. It is mentioned, however, that in the case of some phyla (e.g., Actinobacteria and Bacteroidetes) microbial community composition might also be significantly correlated with other soil environmental variables (e.g., carbon content and availability). At the same time, the diversity of the soil bacterial community has a crucial effect on nutrient (including nitrogen) cycling. Alteration of soil microbial community composition caused by nitrogen content and availability is likely to significantly affect soil organic matter cycling.

The Spearman’s rank correlation coefficient was used to estimate a correlation of alpha-diversity parameters of the microbiome with some physical–chemical parameters of soils (e.g., pH, TOC, nitrogen content, C/N ratio, fine earth content) and basal respiration rate (Table 5).

The pH values were found to be in significant positive correlation with Simpson’s index (R = 0.422). Phylogenetic diversity was found in significant positive correlation with TOC (R = 0.328) and nitrogen content (R = 0.509). Nitrogen content was also found to be positively correlated to OTU number (R = 0.436). Basal respiration and fine earth content was found in significant positive correlation with phylogenetic diversity (R = 0.552 and 0.691).

Our data on significant correlations between alpha-diversity indices and soil parameters (pH, nitrogen concentration and availability) are consistent with those previously reported for different polar regions [25,72,74]. Previous research showed that not only soil moisture, but also pH, significantly affect microbiome composition and diversity in Antarctic environments [22]. Authors showed a clear correlation of increased abundance of several phylum—Cyanobacteria, Gamma- and Deltaproteobacteria, Bacteroidetes and Gemmatimonadetes—with water availability and increased pH levels [2,75]. The diversity of Antarctic microorganisms has been reported to drop significantly along moisture gradients [76,77,78].

Our results suggest that the fluctuations in the taxonomic structure of the soil microbiome might be explained by the total organic carbon and nitrogen content, and to a lesser extent by the soil pH range. Results of previous studies also revealed that soil pH and nitrogen content are the most important contributors to the variation in soil microbial communities, and are considered good predictors of bacterial community composition [60,72,73]. The pH range has been described previously as the main environmental factor in determining soil microbial community composition not only across biomes but also for individual soil types [73,74]. The authors explained the strong influence of pH on the bacterial community composition by the narrow pH ranges optimal for the growth of bacteria. It is also mentioned; however, that in some cases (Actinobacteria and Bacteroidetes), microbial community composition might also be significantly correlated with other soil environmental variables (i.e., carbon content and availability). At the same time, the diversity of the soil bacterial community has a substantial effect on nutrient (including nitrogen) cycling. Alteration of soil microbial community composition caused by nitrogen content and availability is likely to affect soil organic matter cycling. Nutrient availability and moisture content significantly affect the microbial community structure, which was proved by several studies in dry mineral Antarctic soils [75,76,77]. In our study, we identified some representatives of phototrophic microorganisms (Cyanobacteria and Chloroflexi) as well as chemolithotrophic/chemoorganotrophic (Nitrospirae). These groups provide the basis for efficient ecosystem developments, since they take part in primary production.

A role of pH and nutrient content has been discussed in regards of altering the soil microbial communities of Antarctica. However, recent studies concluded different predominant environmental factors affecting soil microbiome. Despite some of them proving that soil microbial communities are mostly affected by carbon, nitrogen and moisture [59], other research reported the main role of K, C, Ca and moisture to affect the distribution and structure of microbial communities in Antarctic Dry Valley [79]. On the other hand, Newsham et al. [80] suggested that nutrient levels have only a minor effect on the bacterial community composition.

A significant correlation found between fine earth and microbial phylogenetic diversity further provides evidence of the vital role of gravel pavement, which creates favorable conditions for bacteria development in the harsh environments of Eastern Antarctica. Gravel pavement “shelters” the sub-surface horizons from dehydration, UV-radiation and strong winds, but simultaneously they are close to the surface, reachable by light and moisture from snow patches and well-insulated. This provides necessary conditions for fine earth formation and soil development.

Selected soil physical–chemical parameters discussed above explained the majority of the variation of the soil bacterial community. However, the role of other factors (such as influence of trace metals content, plant characteristics and productivity, etc.) could not be totally underestimated. Moreover, results of our study also prove the idea that some bacterial species could be introduced into Antarctic soils by human activities [81].

Whereas soil microbial communities have been the subject of detailed study in temperate environments, little information have been published for polar environments. The Antarctic soil microbiome is of particular interest, since comprehensive study of microbial diversity and its effects on the functioning and stability of vulnerable Antarctic ecosystems, as well as their components (e.g., carbon dynamics controlled by the microbiome), remain poorly understood.

## 4. Conclusions

Antarctic soil microbial communities are closely related to the environmental conditions. Therefore, a comprehensive assessment of its structure and dynamics could serve as an effective environmental indicator. According to climatic models, further climate change could lead to expansion of ice-free areas in Antarctica, thus supporting soil development as well as the changing of microbial communities.

Results of soil physical–chemical analysis performed in our study coincided with the idea that lack of moisture serves as the main limiting factor of both soil formation and development, rather than changes in temperature.

Taking into account weak profile development, predominance of coarse fraction in soils and harsh environmental conditions of the studied sites in Larsemann Hills, we obtained quite high bacterial diversity and abundance. The taxonomic analysis of the soil microbiomes revealed 12 major bacterial and archaeal phyla—Proteobacteria, Actinobacteria, Acidobacteria, Chloroflexi, Gemmatimonadetes, Verrucomicrobia, Planctomycetes, Bacteroidetes, Armatimonadetes, Firmicutes, Cyanobacteria and Thaumarchaeota, which constituted the majority (>96% of sequences in amplicon libraries). Our results provide support of the hypothesis that terrestrial ecosystems in the state of initial habitat formation (such as in Eastern Antarctic environments) are usually characterized by diverse but less differentiated microbial communities, compared to analogues with milder climate. Herein, low metabolic activity is believed to be the most important aspect to maintain these diverse communities.

Some specific phyla were also found in sub-surface horizons of the soils investigated, thus providing additional evidence of the crucial role of gravel pavement in creating favorable conditions for both soil and microbiome development.

In this study we also assessed the effect of different soil parameters on the microbial community in harsh environmental conditions of Eastern Antarctica oasis. pH, organic carbon and nitrogen, as well as fine earth content, were revealed as the most accurate predictors of soil bacterial community composition.

Results of our study; thus, provide new insights on environmental drivers of soil microbial communities as well as their characteristic structural and functional diversity in harsh environments of Eastern Antarctica, where detailed investigations on soil microbiology are still rare. Our results, moreover, could be useful for the purposes of environmental risk assessment and effective conservation of fragile Antarctic ecosystems.

## Figures and Tables

**Table 1:** Climatic characteristics of Larsemann Hills, Eastern Antarctica [27].

Coordinates	MAAT, °C	MAST, °C	MAGT, °C	Mean Annual Wind Speed, m s^−1^	Annual Precipitation, mm
*Larsemann Hills*					
S 69°40′23.2″ E 76°34′21.2″	−9.6	−10.4	−9.1	6.7	250

MAAT—Mean annual air temperature; MAST—Mean annual surface temperature; MAGT—Mean annual ground temperature.

**Table 2:** Geographical and soil description of investigation sites.

Site	Geographical Coordinates	Location and Horizon Description	Soil Name (WRB)
**Anthropogenically-Affected Sites**			
Progress 1	69°22′39.7″S 76°23′15.9″E	Area between office and residential buildings; surface horizon	Hyperskeletic Turbic Technosol
Progress 2	69°24′02.6″S 76°24′05.2″E	Vicinities of field base “Progress-3”, wet valley, surface horizon	Hyperskeletic Lithic Leptosol
**Near-Natural Sites**			
Lars 1 (0–1 cm)	69°23′11.4″S 76°23′16.7″E	Surface “algal–mineral” horizon, wet valley of Larsemann Hills	Hyperskeletic Leptosol (Lithic)
Lars 1 (1–5 cm)		Moss–lichen–algae horizon in bottom of the wet valley of Larsemann Hills	
Lars 2 (0–1 cm)	69°23′45.1″S 76°21’08.7″E	Surface coarse horizon under “gravel pavement” in flank of the valley with ephemeral water supply from melting snow patches	Hyperskeletic Leptosol (Lithic, Gleyic)
Lars 2 (1–5 cm)		“Algal–mineral” mineral horizon	
Lars 2 (15–25 cm)		Transitional (BC) horizon, with stagnic and oximorphic features	
Lars 3	69°23′12.9″S 76°19′26.6″E	Gravel pavement in high “dry” flank of the valley	Hyperskeletic Leptosol (Lithic)

**Table 3:** Soil physical–chemical parameters ± SD.

Site	pH_H2O_	pH_KCl_	TOC, %	N, %	C:N	Basal Respiration, mg g^−1^ day^−1^	Fine Earth (<2 mm), %
**Anthropogenically-Affected Sites**							
**Progress 1**	5.85 ± 0.53	5.23 ± 0.42	0.87 ± 0.10	0.083± 0.005	10.48 ± 1.27	0.009 ± 0.001	18.99 ± 1.23
**Progress 2**	5.32 ± 0.56	4.89± 0.32	0.76 ±0.16	0.062 ±0.005	12.26 ± 2.09	0.008 ± 0.001	21.91 ± 1.97
**Near-Natural Sites**							
**Lars 1** **(0–1 cm)**	6.42 ± 0.56	5.76 ± 0.45	0.89 ± 0.12	0.08 ± 0.010	11.13 ± 1.56	0.010 ± 0.002	21.78 ± 1.86
**Lars 1** **(1–5 cm)**	6.01 ±0.43	5.54 ± 0.31	1.50 ± 0.12	0.12± 0.01	12.50 ± 1.23	0.101 ± 0.006	31.98 ± 1.98
**Lars 2** **(0–1 cm)**	6.21 ± 0.42	5.86 ± 0.23	0.98 ± 0.12	0.06 ± 0.01	16.33 ± 1.86	0.009 ± 0.001	22.03 ± 1.54
**Lars 2** **(1–5 cm)**	5.98 ± 0.32	5.54 ± 0.27	1.65 ± 0.17	0.15 ± 0.03	10.84 ± 0.87	0.150 ± 0.010	29.87 ± 1.50
**Lars 2** **(15–25 cm)**	6.03 ± 0.24	5.65 ± 0.23	0.54 ± 0.08	0.04 ± 0.01	13.50 ± 1.24	0.006 ± 0.001	19.87 ± 1.65
**Lars 3**	6.04 ± 0.21	5.76 ± 0.21	0.87 ± 0.10	0.08 ± 0.02	10.88 ± 1.26	0.007 ± 0.001	21.09 ± 1.78

**Table 4:** Alpha-diversity parameters of investigated soils microbiomes.

Site	OTUs	PD_Whole_Tree	Simpson Index	Shannon Index
**Anthropogenically-Affected Sites**				
**Progress 1**	504 ± 32	40 ± 3.5	0.985 ± 0.12	5.02 ± 0.78
**Progress 2**	760 ± 28	58 ± 4.1	0.981 ± 0.15	5.31 ± 0.85
**Near-Natural Sites**				
**Lars 1 (0–1 cm)**	566 ± 26	45 ± 3.5	0.984 ± 0.17	5.10 ± 1.02
**Lars 1 (1–5 cm)**	513 ± 25	46 ± 5.2	0.967 ± 0.11	4.85 ± 0.65
**Lars 2 (0–1 cm)**	476 ± 22	40 ± 4.2	0.981 ± 0.14	4.85 ± 0.98
**Lars 2 (1–5 cm)**	590 ± 29	46 ± 6.1	0.959 ± 0.15	4.75 ± 0.76
**Lars 2 (15–25 cm)**	310 ± 21	28 ± 5.3	0.945 ± 0.23	4.02 ± 0.73
**Lars 3**	403 ± 22	41.5 ± 4.5	0.979 ± 0.25	4.63 ± 0.56

**Table 5:** Correlation matrix between soil parameters and microbial alpha-diversity indices.

	pH	TOC	N	C/N	Basal Respiration	Fine Earth	OTUs	Shannon	Simpson	PD_Tree
**pH**	—									
**TOC**	0.273	—								
**N**	−0.034	0.758 ***	—							
**C/N**	0.285	−0.093	−0.588 **	—						
**Basal Respiration**	0.176	0.876 ***	0.806 ***	−0.242	—					
**Fine Earth**	0.148	0.757 ***	0.522 **	0.258	0.723 ***	—				
**OTUs **	−0.244	0.245	0.436 *	−0.312	0.555 **	0.397	—			
**Shannon**	−0.056	0.032	0.156	−0.101	0.283	0.163	0.795 ***	—		
**Simpson**	0.422 *	0.263	0.267	0.052	0.271	0.207	0.368	0.688 ***	—	
**PD_tree**	−0.167	0.358 *	0.509 *	−0.094	0.552 **	0.691 ***	0.848 ***	0.677 ***	0.391	—

* *p* < 0.05, ** *p* < 0.01, *** *p* < 0.001.
